# Neurogranin in cerebrospinal fluid as a marker of synaptic dysfunction in hip fracture patients with delirium: a multicentre cross-sectional study

**DOI:** 10.1136/bmjopen-2024-097579

**Published:** 2025-11-11

**Authors:** Mathias N P Hella, Nathalie B Halaas, Hogne Soennesyn, Anne K Bergland, Hanne B Hetland, Kaj Blennow, Henrik Zetterberg, Audun O Vik-Mo, Ane-Victoria Idland, Christian T Pollmann, Marius Myrstad, Bjørn E Neerland, Dag Aarsland, Leiv O Watne

**Affiliations:** 1 Department of Clinical Medicine, University of Bergen, Bergen, Norway; 2 Centre for Age-Related Medicine, Stavanger University Hospital, Stavanger, Norway; 3 Oslo Delirium Research Group, Institute of Clinical Medicine, University of Oslo, Oslo, Norway; 4 Department of Research, Stavanger University Hospital, Stavanger, Norway; 5 Department of Psychiatry and Neurochemistry, Institute of Neuroscience & Physiology, the Sahlgrenska Academy, University of Gothenburg, Goteborg, Västra Götaland, Sweden; 6 Clinical Neurochemistry Laboratory, Sahlgrenska University Hospital, Mölndal, Sweden; 7 Paris Brain Institute, ICM, Pitié-Salpêtrière Hospital, Sorbonne University, Paris, Île-de-France, France; 8 Neurodegenerative Disorder Research Center, Division of Life Sciences and Medicine, and Department of Neurology, Institute on Aging and Brain Disorders, University of Science and Technology of China, Hefei, Anhui, China; 9 UCL, UK Dementia Research Institute, London, England, UK; 10 UCL Institute of Neurology, UCL Department of Neurodegenerative Disease, London, UK; 11 Hong Kong Center for Neurodegenerative Diseases, Hong Kong, People's Republic of China; 12 Wisconsin Alzheimer’s Disease Research Center, University of Wisconsin School of Medicine and Public Health, University of Wisconsin-Madison, Madison, WI, USA; 13 Department of Anesthesiology, Akershus University Hospital, Lorenskog, Norway; 14 Department of Orthopedic Surgery, Akershus University Hospital, Lorenskog, Lørenskog, Norway; 15 Department of Internal Medicine, Bærum Hospital, Vestre Viken Hospital Trust, Drammen, Norway; 16 Department of Geriatric Medicine, Oslo University Hospital Ullevaal, Oslo, Norway; 17 Department of Old Age Psychiatry, King’s College London Institute of Psychiatry Psychology & Neuroscience, London, UK; 18 Department of Geriatric Medicine, Akershus University Hospital, Lorenskog, Norway

**Keywords:** Delirium, Dementia, Cognition

## Abstract

**Abstract:**

**Objectives:**

Neurogranin (Ng) has a role in synaptic plasticity and is considered a biomarker of synaptic dysfunction, a process hypothesised to be important in delirium. Few studies examining Ng in delirium exist, with mixed findings. This study aimed to investigate associations between cerebrospinal fluid (CSF) Ng concentrations and delirium in acutely admitted hip fracture patients.

**Design:**

Cross-sectional study.

**Setting:**

Acutely admitted orthopaedic patients with hip fracture recruited from four participating hospitals in eastern Norway, representing secondary and tertiary care settings.

**Participants:**

This study included 392 hip fracture patients. All admitted hip fracture patients operated in spinal anaesthesia were, regardless of age, considered for inclusion.

**Methods:**

An in-house ELISA was used to measure CSF Ng concentration in patients acutely admitted with a hip fracture (n=392). Delirium status was evaluated daily according to The Diagnostic and Statistical Manual of Mental Disorders, Fifth Editions criteria independently by two experienced geriatricians. A value > 3.44 on The Informant Questionnaire on Cognitive Decline in the Elderly was used as a surrogate marker of probable dementia.

**Results:**

180 patients (46 %) developed delirium and 70% of these had dementia. CSF Ng concentration did not differ significantly between those with and without delirium (176 pg/mL vs 164 pg/mL), with an estimated difference in medians of 12 (95% CI −5.8 to 29.8), p=0.185. Analyses adjusted for age, gender and dementia status did not show a statistically significant difference in Ng concentrations between the patients.

**Conclusions:**

We did not find an association between delirium and CSF concentrations of Ng. This could imply that synaptic dysfunction and degeneration, involving Ng, are not key processes in the development of delirium. Further studies on other synaptic proteins are warranted to better explore synaptic dysfunction’s potential role in the pathophysiology of delirium.

**STRENGTHS AND LIMITATIONS OF THIS STUDY**

- The study included a robust sample size for a cerebrospinal fluid biomarker study in delirium and used a structured system for delirium diagnosis by two independent geriatricians.
- Studying neurogranin in cerebrospinal fluid is a strength as this is considered to better reflect the processes of the brain than blood biomarkers.
- Patients undergoing acute surgery are not necessarily comparable to other patient groups—in terms of generalisability regarding delirium pathophysiology.
- Given the study design baseline cognitive testing was not possible and we used the Informant Questionnaire on Cognitive Decline in the Elderly as a surrogate marker for dementia which, although validated and much used, is still a limitation with our study.
- We have examined one of many synaptic proteins at one timepoint, which means we cannot rule out the importance of other synaptic proteins in the pathophysiology of delirium.

## Introduction

 Delirium is a serious complication of acute illness with risk factors including old age and surgery.^1^ Delirium in hip fracture patients is very common, affecting 35%–65%,^2^ and is associated with complications, institutionalisation and 6-month mortality.^3^ Negative outcomes associated with delirium, including mortality, are also observed in other patient care settings, such as medical patients^4^ and critically ill patients.^5^ Delirium pathophysiology is not well understood, but key processes likely include neuroinflammation, metabolic changes in the brain and/or an imbalance in central nervous system (CNS) neurotransmitters.^1 6 7^

Delirium and dementia have a complex interrelationship, as people with dementia have a higher risk of developing delirium, and delirium is a risk factor for developing dementia.^8^ A recent study showed delirium to be associated with both worse cognition at follow-up and increased concentrations of neurofilament light chain, a marker of neuronal injury.^9^ Indeed, cerebrospinal fluid (CSF) biomarkers may help provide insight into delirium pathophysiology,^10 11^ by exploring associations of biomarkers with specific functions in the CNS with delirium.

In 1990, a protein, p17, now named neurogranin (Ng), was identified in ‘granule-like structures’ in the CNS.^12^ It is a protein kinase C substrate—a postsynaptic protein.^12^ Protein kinases are important enzymes which alter the function of other proteins in the nervous system.^13^ Ng binds calmodulin, which raises intracellular Ca^2+^ and enhances learning, memory and synaptic plasticity.^14 15^ Synaptic dysfunction is implicated in the development of various diseases, including Alzheimer’s disease (AD).^16^ Baseline concentrations of Ng in CSF have been found increased in patients with mild cognitive impairment and in AD patients compared with cognitively unimpaired adults,^17^ likely indicating synaptic dysfunction or loss. Autopsy studies of brain tissue have, however, demonstrated a decrease of Ng in remaining synapses of Alzheimer’s patients compared with healthy adults, potentially explaining the previously observed increase in CSF Ng.^18^

The two previous studies examining Ng in delirium, known to us, show conflicting results. Ng is expressed in brain regions that could be involved in the development of delirium, such as the cortex and hippocampus,^19^ and synaptic dysfunction or degeneration has been hypothesised to be of importance in the pathophysiology of delirium.^20^ Several conditions associated with delirium, such as an infection or a hip fracture, induce an inflammatory response in the body, in line with the neuroinflammatory hypothesis of delirium pathophysiology. This inflammatory response also involves the CNS where it induces dysfunction in neurons and is suggested to affect synaptic plasticity.^7^ In mice, bacterial lipopolysaccharide induced acute and fluctuating cognitive dysfunction and neuropathological synaptic loss, indicating that synaptic dysfunction or loss may be of importance in delirium pathophysiology.^19^ Interestingly, in a study among patients with infection and delirium, compared with patients with infection and no delirium, Peters van Ton *et al* reported lower concentrations of synapse-related proteins in CSF.^21^ This study did not include Ng and to our knowledge only two studies have specifically examined Ng concentrations in patients with delirium. Wanderlind *et al* studied blood concentrations of Ng in 97 critically ill patients, 47 of them with delirium. They found higher concentrations of Ng and interleukin-1 beta in the delirium group on the day of intensive care unit admission, and at delirium diagnosis, compared with the control group.^22^ In the only previously known CSF study of Ng in delirium, Halaas *et al* did not find an association between delirium in hip fracture patients (n=70 patients with delirium vs n=58 patients without delirium) and CSF Ng concentrations.^23^ In summary, the previous studies on Ng in delirium are few, with relatively small sample sizes and conflicting results, advocating the need for a larger biomarker study exploring the association of Ng with delirium, which is the aim of this study.

## Materials and methods

### Participants

The study cohort comprised hip fracture patients, with and without delirium and with and without dementia.

The hip fracture patients (n=392) included in this study participated in a multicentre study with four participating hospitals, representing secondary and tertiary care settings in Norway between 2016 and 2020. Inclusion criteria: all admitted hip fracture patients who underwent surgery in spinal anaesthesia, without age limitations. We had no exclusion criteria. Written informed consent was obtained from all study participants, or from their proxies if the participants were unable to consent. No power calculations were performed as all available CSF samples were analysed. Cognitive status prior to the hip fracture was assessed using the Informant Questionnaire on Cognitive Decline in the Elderly (IQCODE), an informant-based questionnaire, with > 3.44 as a cut-off indicating possible dementia.^24^ In cases of missing IQCODE (n=36), the dementia status was decided based on all the available information from the hospital records, from all healthcare personnel.

### Delirium diagnosis

The patients were evaluated daily for delirium preoperatively and until the fifth postoperative day for patients without delirium or until discharge for patients with delirium, according to The Diagnostic and Statistical Manual of Mental Disorders, Fifth Edition criteria (DSM-5).^25^ The patient’s level of arousal was scored using the Richmond Agitation Sedation Scale,^26^ and the Observational Scale of Level of Arousal.^27^ Trained study nurses performed the interviews with the patients. Two geriatricians (LOW and BEN) independently considered all the information available for each patient to decide if the DSM-5 criteria^25^ were met. Any disagreements were solved through discussion and consensus obtained. The patients were further classified into subgroups: no delirium, subsyndromal delirium, incident delirium (no delirium at the time of CSF sampling but developed it later) and prevalent delirium (delirium at CSF sampling). Subsyndromal delirium was defined as patients having evidence of acute cognitive changes from baseline, in addition to one of the following: altered arousal, attentional deficits, other cognitive changes, delusions or hallucinations, thus fulfilling some, but not all the criteria for a delirium.

### CSF sampling and biochemical analyses

CSF was collected in conjunction with spinal anaesthesia before administration of anaesthetic agents. CSF was collected in polypropylene tubes. Samples were centrifuged at 2000*g*, and supernatants aliquoted and stored at −80°C. Samples were then sent on dry ice for biochemical analyses of Ng at Sahlgrenska University Hospital (Mölndal, Sweden). An in-house ELISA was used to measure CSF Ng concentration in the samples, described in more detail in a previous publication.^28^

### Statistical methods

To present patient characteristics we used standard descriptive statistics. Normality for continuous variables was evaluated by the distribution of the data’s histogram, the Shapiro-Wilk and Kolmogorov-Smirnov tests. Age was not normally distributed and thus reported as median (IQR). Sex and dementia status were reported as number (percent). For group comparisons, continuous variables were analysed using Mann-Whitney U test and categorical variables were analysed using χ^2^ statistics.

Ng concentrations were not normally distributed, we thus report the variables as median (IQR). Observed distributions in delirium groups were presented in violin plots and compared using Mann-Whitney and Kruskal-Wallis tests. Correlation between Ng concentration and age was calculated with Spearman’s ρ.

Further, we used quantile regression to estimate differences in medians between groups and to adjust for sex, age and dementia. Supplementary results are presented for subgroups of dementia status. Estimated differences are presented with 95% CIs. Unlike linear regression, which estimates means and can be sensitive to outliers, quantile regression estimates conditional quantiles such as the median. This makes it particularly suitable for data that are skewed or contain outliers, as it does not assume normality and provides more robust estimates of central tendency.^29^

Missing data: All available cases were used in each analysis.

A two-tailed p-value of <0.05 was considered statistically significant. The statistical analyses were performed in IBM SPSS Statistics V.26 software. The violin plots were created using vioplot 0.4.0 in R V.433.

### Patient and public involvement

None.

## Results

### Delirium overall and baseline characteristics

Of the 392 hip fracture patients, 180 (46%) experienced delirium during the hospitalisation. Of these, delirium developed before surgery in 87 patients (prevalent delirium). The remaining patients (n=93) were free from delirium before surgery but developed delirium postoperatively (incident delirium). Compared with those without delirium, patients with delirium were older (median 86 years vs 77 years, p<0.001) and more had probable dementia (71% vs 15%, p<0.001). There was no significant difference in the delirium and no delirium group with respect to sex (table 1). Subsyndromal delirium was observed in 7.7% of the patients (n=30).

**Table 1:** Population characteristics

	All hip fracture patients (n=392)	No delirium (n=212)	Delirium (n=180)	P value
Age	82 (74–88)	77 (70–85)	86 (80–91)	<0.001
Female sex	266 (68)	142 (67)	124 (69)	0.69
Dementia*	159 (41)	32 (15)	127 (71)	<0.001

* Dementia defined by a score of 3.44 or above on the Informant Questionnaire on Cognitive Decline in the Elderly (IQCODE). In case of missing IQCODE (n=36) dementia status was based on information from the hospital record. Results are given as median (IQR), or n (%). Mann-Whitney U test was used for comparison of age. Categorical variables were analysed using χ2 statistics.

### Association between delirium status and neurogranin

**Figure 1:**
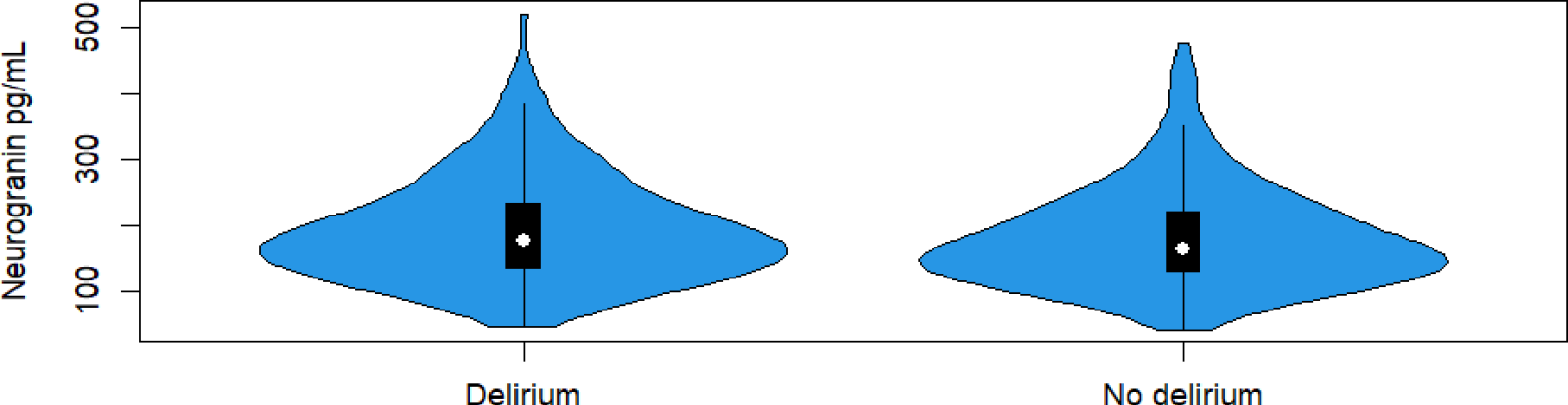
Violin plot showing a comparison of neurogranin in picograms per millilitre (pg/mL) in patients with delirium (n=180) and without delirium (n=212), by Mann-Whitney U-test showing no statistically significant difference between the groups, p-value 0.087.

Figure 1 visualises Ng concentrations in a violin plot, comparing patients with and without delirium. Using quantile regression (table 2), we found no difference in CSF Ng concentrations between hip fracture patients with and without delirium with median (IQR) 176 (134–235) versus 164 (130–219), with an estimated difference in medians of 12 (95% CI −5.8 to 29.8), p=0.185. Adjusting for probable dementia, age and sex did not change this (table 2).

**Table 2:** Comparison of cerebrospinal fluid (CSF) concentrations of neurogranin (Ng) for patients with and without delirium

				Unadjusted		Adjusted	
	All hip fracture patients (n=392); median (IQR)	No delirium (n=212); median (IQR)	Delirium (n=180); median (IQR)	Difference (95% CI)	P value	Difference* (95% CI)	P value
CSF Ng, pg/mL	170 (133–225)	164 (130–219)	176 (134–235)	12 (−5.8 to 29.8)	0.185	9 (−12.6 to 30.6)	0.414

Results reported as estimated difference in medians by quantile regression.

* Adjusted for age, sex, and dementia status.

CI, confidence interval; IQR, interquartile range.

### Delirium subgroup analysis of neurogranin concentrations

**Figure 2:**
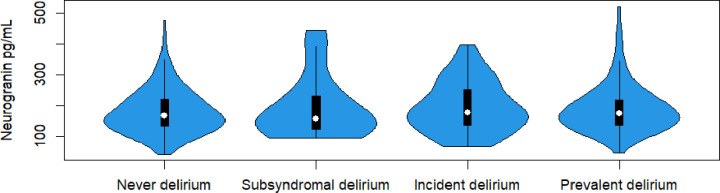
Violin plot showing a comparison of neurogranin in picograms per millilitre (pg/mL) in different delirium subgroups: never delirium (n=177), subsyndromal delirium (n=30), incident delirium (n=93) and prevalent delirium (n=87). We found no statistically significant difference between the groups by Kruskal-Wallis test, p value 0.461. Patients missing delirium subclassification (n=5) were not included in the analysis.

We explored whether the concentrations of Ng differed between subgroups of delirium (no delirium, subsyndromal delirium, incident and prevalent delirium). We did not find any differences in CSF Ng concentration across delirium subgroups (p=0.46, figure 2). We analysed the data by dementia status and found no significant difference in CSF Ng concentration when comparing patients with and without delirium (online supplemental table). Adjusting for age and sex did not change this.

### Supplementary results

We found no statistically significant differences in the CSF Ng values when comparing patients with (median 174 pg/mL (IQR 133–223)) and without (167 pg/mL (IQR 131–226), p=0.48) probable dementia.

A positive correlation (r=0.20, p=<0.001) was found between age and Ng values.

## Discussion

In our study of acutely admitted hip fracture patients, we did not find an association between delirium and CSF Ng concentrations. These results are in line with a prior smaller CSF study^23^ and, taken together, this suggests that synaptic dysfunction, involving Ng, may not be an important part of delirium pathophysiology.

Studies have demonstrated higher concentrations of CSF Ng in mild cognitive impairment (MCI) and Alzheimer’s disease (AD) patients, possibly indicating synaptic injury or dysfunction, and a correlation between Ng concentration and progression of cognitive decline.^17 30^ One of these studies^30^ found intra-individual levels of Ng to not increase in later stages of MCI or AD, but in cognitively normal adults, which could indicate that increased Ng concentrations reflect presymptomatic injury or dysfunction to the synapse. Brain tissue autopsy studies have shown decreased concentrations of Ng in AD patients compared with healthy controls, likely indicating loss of synapses, and potentially explaining the increase in CSF Ng.^18^ Halaas *et al* did not find a difference in CSF Ng in hip fracture patients with and without delirium,^23^ which is in line with our findings. Lower concentrations of synapse-related proteins, such as neuroblastoma suppressor of tumourigenicity, thy-1 membrane glycoprotein, neuronal cell adhesion molecule, tumour necrosis factor receptor super family member 21, neurocan core protein and brevian core protein in CSF have, however, been demonstrated in a study of 15 patients with infection and delirium, compared with patients with infection and no delirium.^21^ The authors also found lower concentrations of synapse-related proteins in AD patients and interpreted their findings as potentially pointing towards shared pathophysiological mechanisms in patients with infection who have developed delirium and patients with AD. This study did not examine Ng specifically but studied other proteins that have a role in synaptic transmission and plasticity, and the findings could indicate that synaptic proteins are downregulated in delirium. An alternative hypothesis for our findings of no association between delirium and Ng could be that in our patients, with advanced age and some with probable dementia, Ng concentrations have already been altered due to pre-existing synaptic injury, loss and degeneration. This could make differences in Ng concentrations more difficult to demonstrate in this patient population.

Blood-brain barrier (BBB) leakage is hypothesised to be relevant for some patients with delirium.^31^ Ng is a small molecule that may cross the BBB, possibly more so when the barrier is damaged. Taylor *et al* found postoperative delirium to be associated with a breakdown in the BBB and hypothesise that peripheral inflammation induces this breakdown and leads to an anti-inflammatory response in the CNS and delirium development through synaptic suppression.^32^ As delirium pathophysiology is likely multifactorial with complex interactions, this could be a potential model linking BBB leakage to neuroinflammation and synaptic dysfunction measured by Ng. Wanderlind *et al* found blood concentrations of Ng to be higher in the delirium group both on the day of ICU admission and on the day of delirium diagnosis compared with the no delirium group.^22^ However, no correlation between blood and CSF Ng was found in patients with cerebral stroke^33^ and in a study of AD patients and controls.^28^ These studies demonstrate the importance of studying delirium using CSF.

### Strengths and limitations

Strengths of our study include the study sample size, which is large for a CSF biomarker study in delirium. Studying Ng concentrations in CSF is important considering other studies that have shown no correlation between blood and CSF Ng.^28^ Our method of diagnosing delirium, based on thorough daily examination with validated tests for arousal and attention, is also a strength.

Our study also has several limitations. Using IQCODE as a surrogate marker of probable dementia is one limitation. Even though IQCODE has shown good sensitivity in identifying people with dementia,^24^ it is not a formal diagnosis by cognitive testing. It is also important to remember that patients undergoing acute surgery are not necessarily comparable to other patient groups—in terms of generalisability regarding delirium pathophysiology.^2^ The acute trauma and systemic inflammatory reaction triggered by a hip fracture^34^ are characteristics not shared by all patients experiencing delirium. We also want to highlight that we have studied one of many synaptic proteins at one timepoint. It could thus be of interest also to specifically examine the concentrations of other synaptic proteins—for example, synapse-related proteins that have been shown to be downregulated in delirium.^21^ Ideally, biomarker samples should be collected at several timepoints to establish a baseline and register any eventual changes from this baseline after development and resolvement of delirium. It is possible to collect repeated blood samples from acutely admitted patients, but unfortunately both practically and ethically, it is extremely difficult with repeated CSF sampling over time in such patient groups.

Our study has several potential confounders. The median age in our patients with delirium was high—86 years and we found a positive correlation between age and Ng. A previous study, with repeated CSF sampling, found CSF Ng increased over time in cognitively normal participants, whereas patients with MCI or AD had increased baseline concentrations of Ng and no increase over time in repeated sampling.^30^ Casaletto *et al* similarly found higher Ng concentrations in CSF with older age in cognitively normal patients.^35^ Adjusting for age and sex in our statistical analysis did, however, not affect the results. Dementia status is also of relevance, as discussed previously, we did, however, not find any statistically significant differences in the concentrations of Ng in patients with and without dementia. A total of 127 patients in our study with delirium also had dementia. Norwegian hip fracture patients are generally operated with a mean waiting time from trauma of 22 hours^36^ and it could theoretically take longer for CSF Ng to rise. Even so, a study on stroke patients found significantly higher CSF Ng values in patients with stroke 9 hours after the onset of stroke symptoms.^33^ This could indicate that Ng increases rapidly in CSF, and if Ng plays a role in delirium pathophysiology, we would have expected to see differences between groups in our study.

## Conclusion

In summary, our findings do not support synaptic dysfunction, involving Ng, as an important mechanism in the pathophysiology of delirium. As Ng has been demonstrated to be an important protein in synaptic plasticity, this was a relevant biomarker to study in relation to delirium pathophysiology. Our study showed no association between Ng and delirium in hip fracture patients which could indicate that the pathophysiological processes underlying delirium are unlikely to primarily involve postsynaptic mechanisms. We have, however, only studied one of many synaptic proteins at one timepoint, meaning further studies on other synaptic proteins, such as panels of different presynaptic biomarkers, are warranted to better explore synaptic dysfunction’s potential role in the pathophysiology of delirium.

## Supplementary material

10.1136/bmjopen-2024-097579 online supplemental file 1

## Data Availability

Data are available upon reasonable request.
