# Supplementary material for: Neurogranin in cerebrospinal fluid as a marker of synaptic dysfunction in hip fracture patients with delirium: a multicentre cross-sectional study
Source: BMJ Open. 2025 Nov 11;15(11):e097579. doi: 10.1136/bmjopen-2024-097579 (PMC12606483; doi:10.1136/bmjopen-2024-097579)
Supplement: online supplemental file 1 [file bmjopen-15-11-s001.docx]

**Supplements:**

Mathias N.P. Hella et al, “Neurogranin in delirium”.

**Table 1:** Comparison of the median values with interquartile range of Ng concentrations in delirium subgroups and by dementia status. Estimated differences in medians were calculated by quantile regression, comparing the different subgroups against never delirium as a reference group, not corrected for age and sex.

|  | **Never delirium** | **Subsyndromal delirium** | | **Incident delirium** | | **Prevalent delirium** | |
| --- | --- | --- | --- | --- | --- | --- | --- |
|  | Median  (IQR) | Median  (IQR) | Difference  (95% CI) | Median  (IQR) | Difference  (95% CI) | Median  (IQR) | Difference  (95% CI) |
| **All patients** | 166  (133, 220) | 156  (120, 238) | -13.0  (-48.0, 22.0) | 157  (134, 257) | 11.0  (-11.7, 33.7) | 175  (134, 221) | 9.0  (-14.2, 32.2) |
| **Dementia** | 176  (149, 255) | 140  (109, 181) | -44.0  (-103.4, 15.4) | 179  (140, 240) | 0.0  (-42.9, 42.9) | 171  (133, 214) | -6.0  (-48.2, 36.2) |
| **No dementia** | 176  (132, 219) | 173  (135, 247) | 11.8  (-35.9, 59.5) | 166  (126, 291) | 1.0  (-35.2, 37.2) | 193  (135, 247) | 11.8  (-35.9, 59.5) |

Patients missing delirium subclassification (n=5) were not included in the analysis.
