# Supplementary material for: Neurogranin in cerebrospinal fluid as a marker of synaptic dysfunction in hip fracture patients with delirium: a multicentre cross-sectional study
Source: BMJ Open. 2025 Nov 11;15(11):e097579. doi: 10.1136/bmjopen-2024-097579 (PMC12606483; doi:10.1136/bmjopen-2024-097579)
Supplement: Supplementary file 2 [file bmjopen-2024-097579.reviewer_comments.pdf]

PEER REVIEW HISTORY

BMJ Open publishes all reviews undertaken for accepted manuscripts. Reviewers are asked to complete a checklist review form and are provided with free text boxes to elaborate on their assessment. These free text comments are reproduced below.

ARTICLE DETAILS

Title (Provisional)

Neurogranin in cerebrospinal fluid as a marker of synaptic dysfunction in hip fracture patients with delirium: a multicentre cross-sectional study.

Authors

Hella, Mathias Nikolai Petersen; Halaas, Nathalie Bodd; Soennesyn, Hogne; Bergland, Anne Katrine; Hetland, Hanne Brit; Blennow, Kaj; Zetterberg, Henrik; Osland Vik-Mo, Audun; Idland, Ane-Victoria; Pollmann, Christian Thomas; Myrstad, Marius; Neerland, Bjørn Erik; Aarsland, Dag; Watne , Leiv Otto

---

VERSION 1 - REVIEW

**Reviewer**	**1**
**Name**	**Nitchingham, Anita**
**Affiliation**	**Prince of Wales Hospital and Community Health Services, Aged Care**
**Date**	**19-Feb-2025**
**COI**	

Thank you for the opportunity to review this manuscript. It's well written, easy to follow and I enjoyed reading it.

This study explores the relationship between CSF neurogranin (Ng) and delirium in patients undergoing surgery for hip fracture. The authors found no association between delirium and CSF Ng concentration, concluding that Ng-related synaptic dysfunction may not be a key process in the development of delirium.

The authors have previously published a smaller study with different control groups and similarly found no association between delirium and neurogranin. Additionally, they observed that hip fracture patients had lower CSF-neurogranin concentration than AD patients and cognitively unimpaired older adults after adjusting for age. None-the-less, the current study remains novel, has a large sample size and adds important new knowledge to the field.

I have a few minor suggestions:

Strengths and limitations bullet points:

Given the high prevalence of dementia in this study and its crucial role in interpreting biomarkers in delirium research, I suggest the authors explicitly highlight the limitation related to baseline cognitive testing and dementia diagnosis in this section (e.g. given the study design, baseline cognitive testing and diagnosis of dementia were not possible, but validated surrogate markers were used).

Introduction:

Readers may not be familiar with the relationship between Ng and mild cognitive impairment and dementia. Given the overlap between delirium and dementia, it would be helpful to add one or two sentences explaining this relationship. This will help the readers to conceptualise the findings. This could be added at the end of paragraph 3.

Discussion:

The relationship between age and Ng could be clarified. The authors cite a study in mice showing reduced Ng with age; however, their own data found a positive correlation between age and Ng levels which aligns with other human studies.

Kester et al (doi:10.1001/jamaneurol.2015.1867) found Ng increased overtime in cognitively normal participants (but not MCI or AD).

Casaletto et al (https://doi.org/10.1212/WNL.0000000000004569) found higher Ng concentrations with older age. The discussion should clarify this apparent discrepancy and emphasise the relevance of human data over animal models.

Are there any suggestions for future research? E.g. is there a still a role for studying synaptic dysfunction beyond Ng? e.g. presynaptic proteins.

Minor typographical errors:

• Results section 3.2, 2nd line (NG instead of Ng).

• Discussion, 3rd line of strengths and limitations section said “blood og CSF” – authors to please clarify.

• Figure 1A/1B “Violine” instead of “Violin” plot).

**Reviewer**	**2**
**Name**	**Umoh, Mfon E.**
**Affiliation**	**The Johns Hopkins University School of Medicine**
**Date**	**23-Jul-2025**
**COI**	

Review- Manuscript ID: bmjopen-2024-097579

Overview: The authors completed secondary analysis on data from a multi-center study of hip fracture patients for whom preoperative CSF was collected and analyzed for neurogranin levels and delirium (whether prevalent admission, subsyndromal, or incident). They examine the association between neurogranin (from CSF obtained preoperatively during spinal anesthesia administration), study participants were evaluated daily for delirium using DSM-5 criteria. The premise of the study is to better understand delirium pathophysiology and whether synaptic dysfunction is implicated.

However, in this study they examine 1 synaptic (post) protein, at one time point (preoperatively) and attempt to relate several outcomes to these levels. Given their question of delirium association it may have been clearer if they exclude those with prevalent delirium because that confuses the analysis. Also, the rationale for statistical methods used (quantile regression) should be clearly explained. Our understanding of delirium pathophysiology is very limited, so this is important area of inquiry. There were parts of the manuscript that could be improved (noted throughout pdf comments and highlighted below):

- Abstract: Should include the tension/knowledge gap this work fills- the context of this work is that few studies examining Ng in delirium have shown mixed findings. If this is not included, then the necessity and significance of this analysis is unclear

-Introduction: Prior work informing the study hypothesis should be more clearly reviewed and presented for the audience to better link how synaptic dysregulation may associate with delirium- clearly presenting models that inform hypothesis

- Methods: clarification of when log transformed values were used vs raw. Rationale for statistical analyses with quantile regression and potential limitations with this method.

- 'Probable dementia' from IQCODE should be consistent in language and report how they stratify analyses

- Clarify delirium diagnostic consensus methods

-Discussion: There are more limitations of this work than reported in the discussion and in the strengths and limitations of this study section after the abstract- including having one CSF sample and attempting to understand numerous associations from one time point and evaluating one post synaptic marker.

- The authors do not report how neurogranin levels relate to other delirium biomarkers that have been assessed in this multi-center cohort, as delirium pathophysiology is likely multifactorial, the one marker association with delirium analysis evaluation does not reflect this.

- Conclusions - The significance of this work should be revisited, including future directions and how finding from this study inform a better understanding of delirium pathophysiology

****** The reviewer provided a marked copy with additional comments. Please contact the publisher for full details. ******

VERSION 1 - AUTHOR RESPONSE

Responses to Reviewer #1:

Reviewer: 1
Dr. Anita Nitchingham, Prince of Wales Hospital and Community Health Services
Comments to the Author:

1. Thank you for the opportunity to review this manuscript. It's well written, easy to follow and I enjoyed reading it. This study explores the relationship between CSF neurogranin (Ng) and delirium in patients undergoing surgery for hip fracture. The authors found no association between delirium and CSF Ng concentration, concluding that Ng-related synaptic dysfunction may not be a key process in the development of delirium.

The authors have previously published a smaller study with different control groups and similarly found no association between delirium and neurogranin. Additionally, they observed that hip fracture patients had lower CSF-neurogranin concentration than AD patients and cognitively unimpaired older adults after adjusting for age. None-the-less, the current study remains novel, has a large sample size and adds important new knowledge to the field.

> **A. We thank the reviewer for this encouraging acknowledgment of our work. Your suggestions and corrections have helped us improve the quality of the paper.**

2. I have a few minor suggestions:

Strengths and limitations bullet points:
Given the high prevalence of dementia in this study and its crucial role in interpreting biomarkers in delirium research, I suggest the authors explicitly highlight the limitation related to baseline cognitive testing and dementia diagnosis in this section (e.g. given the study design, baseline cognitive testing and diagnosis of dementia were not possible, but validated surrogate markers were used).

> **A. We agree and have highlighted the lack of a formal dementia diagnosis as a limitation with our study in the strengths and limitations section as an extra bullet point. This section now reads:**

Page 4, line 83-85: Given the study design baseline cognitive testing was not possible. As a surrogate marker for probable dementia, we used the IQCODE. Although this is a validated and much used tool in acute settings, it is still a limitation with our study.

3. Introduction:
Readers may not be familiar with the relationship between Ng and mild cognitive impairment and dementia. Given the overlap between delirium and dementia, it would be helpful to add one or two sentences explaining this relationship. This will help the readers to conceptualise the findings. This could be added at the end of paragraph 3.

A. Thank you for this comment, we agree that this is central and have now added an explanation of this relationship at the end of paragraph 3 of the introduction (page 6). This section now reads:

Page 6, line 114-119: Baseline concentrations of Ng in CSF have been found increased in patients with mild cognitive impairment and in AD patients compared with cognitively unimpaired adults (17), likely indicating synaptic dysfunction or loss. Autopsy studies of brain tissue have however demonstrated decrease of Ng in remaining synapses of Alzheimer's patients compared with healthy adults, potentially explaining the previously observed increase in CSF Ng (18).

4. Discussion:
The relationship between age and Ng could be clarified. The authors cite a study in mice showing reduced Ng with age; however, their own data found a positive correlation between age and Ng levels which aligns with other human studies.
Kester et al (doi:10.1001/jamaneurol.2015.1867) found Ng increased overtime in cognitively normal participants (but not MCI or AD).
Casaletto et al (https://doi.org/10.1212/WNL.0000000000004569) found higher Ng concentrations with older age. The discussion should clarify this apparent discrepancy and emphasise the relevance of human data over animal models.

A. Thank you for this important comment. We agree that the relationship between age and Ng should be clarified. It is correct that our own data (and previous studies) have found a positive correlation between age and Ng in CSF. The study we previously referred to examined brain tissue samples in mice and we have decided to alter this reference as human studies are available and more relevant to our study. The results of higher Ng concentrations with older age are from CSF samples, such as in the study from Calsaletto et al. We have referred to this study and the study from Kester et al in our manuscript. It is also important to mention that brain tissue autopsy studies (Saunders et al 2018, reference 18) have shown decreased concentrations of Ng in AD patients compared to healthy controls, likely indicating loss of synapses, and potentially explaining the increase in CSF Ng previously mentioned. We have tried to clarify this in the discussion of our manuscript. These sections now reads:

Page 14, line 292-294: Brain tissue autopsy studies have shown decreased concentrations of Ng in AD patients compared to healthy controls, likely indicating loss of synapses, and potentially explaining the increase in CSF Ng (18).

Page 16, line 348-354: The median age in our patients with delirium was high- 86 years and we found a positive correlation between age and Ng. A previous study, with repeated CSF sampling, found CSF Ng increased over time in cognitively normal participants, whereas patients with MCI or AD had increased baseline concentrations of Ng and no increase over time in repeated sampling (30). Casaletto *et al* similarly found higher Ng concentrations in CSF with older age in cognitively normal patients (35). Adjusting for age and sex in our statistical analysis did however not affect the results.

5. Are there any suggestions for future research? E.g. is there a still a role for studying synaptic dysfunction beyond Ng? e.g. presynaptic proteins.

A. Thank you for the comment. As we have only studied one of many synaptic proteins, we cannot exclude the possibility that other synaptic proteins e.g. presynaptic, have a potential

role in the pathophysiology of delirium. We have added studies of other synaptic proteins as a recommendation for future research in the conclusion. This section now reads:

Page 17, line 366-374: In summary, our findings do not support synaptic dysfunction, involving Ng, as an important mechanism in the pathophysiology of delirium. As Ng has been demonstrated to be an important protein in synaptic plasticity, this was a relevant biomarker to study in relation to delirium pathophysiology. Our study showed no association between Ng and delirium in hip fracture patients which could indicate that the pathophysiological processes underlying delirium are unlikely to primarily involve postsynaptic mechanisms. We have however only studied one of many synaptic proteins at one timepoint, meaning further studies on other synaptic proteins, such as panels of different presynaptic biomarkers, are warranted to better explore synaptic dysfunction's potential role in the pathophysiology of delirium.

6. Minor typographical errors:
• Results section 3.2, 2nd line (NG instead of Ng).

A. This error is now corrected.

7. Discussion, 3rd line of strengths and limitations section said "blood og CSF" – authors to please clarify.

A. This is corrected to "blood and CSF".

8. Figure 1A/1B "Violine" instead of "Violin" plot).

A. Thank you for the thorough evaluation. We have corrected this error in the revision.

<u>Responses to Reviewer #2:</u>

Reviewer: 2
Dr. Mfon E. Umoh, The Johns Hopkins University School of Medicine
Comments to the Author:

1. Overview: The authors completed secondary analysis on data from a multi-center study of hip fracture patients for whom preoperative CSF was collected and analysed for neurogranin levels and delirium (whether prevalent admission, subsyndromal, or incident). They examine the association between neurogranin (from CSF obtained preoperatively during spinal anaesthesia administration), study participants were evaluated daily for delirium using DSM-5 criteria. The premise of the study is to better understand delirium pathophysiology and whether synaptic dysfunction is implicated. However, in this study they examine 1 synaptic (post) protein, at one time point (preoperatively) and attempt to relate several outcomes to these levels. Given their question of delirium association, it may have been clearer if they exclude those with prevalent delirium because that confuses the analysis. Also, the rationale for statistical methods used (quantile regression) should be clearly explained. Our understanding of delirium pathophysiology is very limited, so this is important area of inquiry.

A. We thank the reviewer for acknowledging the importance of studying delirium pathophysiology and for a thorough revision of our work with important suggestions for improvement. We have chosen to study Ng as it is believed to be a marker of synaptic plasticity. Delirium pathophysiology is not well understood, and synaptic dysfunction has been hypothesized to be of importance in delirium development. We understand that including both patients with prevalent delirium (ongoing delirium at the time of CSF sampling), and

incident delirium (free from delirium at the time of sampling but developed it later) adds to the complexity of our study. However, we will argue to keep both groups in. Since the data on Ng in delirium is very limited, we don't know if it plays any role. Theoretically it could be important in the phase leading up to delirium and/or being a risk factor for delirium. To study that, those with incident delirium is most relevant. But theoretically Ng could also be a marker of ongoing delirium, and if so, samples from patients with prevalent delirium are most valuable.

2. Also, the rationale for statistical methods used (quantile regression) should be clearly explained.

A. We agree that the rationale for using quantile regression was not clearly explained and have elaborated on this in our revision under 2.4 in the methods section. This section now reads:

Page 10, line 203-206: Unlike linear regression, which estimates means and can be sensitive to outliers, quantile regression estimates conditional quantiles such as the median. This makes it particularly suitable for data that are skewed or contains outliers, as it does not assume normality and provides more robust estimates of central tendency (29).

3. Abstract: Should include the tension/knowledge gap this work fills- the context of this work is that few studies examining Ng in delirium have shown mixed findings. If this is not included, then the necessity and significance of this analysis is unclear.

A. Thank you for this comment. We agree and have added the tension/knowledge gap this work fills in the abstract. This section now reads:

Page 2, line 48-52: Objectives: Neurogranin (Ng) has a role in synaptic plasticity and is considered a biomarker of synaptic dysfunction, a process hypothesised to be important in delirium. Few studies examining Ng in delirium exist, with mixed findings. This study aimed to investigate associations between cerebrospinal fluid (CSF) Ng concentrations and delirium in acutely admitted hip fracture patients.

4. Introduction: Prior work informing the study hypothesis should be more clearly reviewed and presented for the audience to better link how synaptic dysregulation may associate with delirium- clearly presenting models that inform hypothesis.

A. We agree and have tried to explain the rationale for examining synaptic dysfunction in delirium in more detail in the introduction. Presenting the work informing the study hypothesis more clearly has in our opinion improved the manuscript and this was an important comment. This section now reads:

Page 6, line 121-143: The two previous studies examining Ng in delirium, known to us, show conflicting results. Ng is expressed in brain regions that could be involved in the development of delirium, such as the cortex and hippocampus (19), and synaptic dysfunction or degeneration has been hypothesized to be of importance in the pathophysiology of delirium (20). Several conditions associated with delirium, such as an infection or a hip fracture, induce an inflammatory response in the body, in line with the neuroinflammatory hypothesis of delirium pathophysiology. This inflammatory response also involves the central nervous system where it is induces dysfunction in neurons and is suggested to affect synaptic plasticity (7). In mice bacterial lipopolysaccharide induced acute and fluctuating cognitive dysfunction and neuropathological synaptic loss,

indicating that synaptic dysfunction or loss may be of importance in delirium pathophysiology (19). Interestingly, in a study among patients with infection and delirium, compared to patients with infection and no delirium, van Ton *et al.*, reported lower concentrations of synapse-related proteins in CSF (21). This study did not include Ng and to our knowledge only two studies have specifically examined Ng concentrations in patients with delirium. Wanderlind et *al.* studied blood concentrations of Ng in 97 critically ill patients, 47 of them with delirium. They found higher concentrations of Ng and IL-1β in the delirium group on the day of ICU admission, and at delirium diagnosis, compared to the control group (22). In the only previously known CSF study of Ng in delirium, Halaas *et al.* did not find an association between delirium in hip fracture patients (n=70 patients with delirium, vs n=58 patients without delirium) and CSF Ng concentrations (23). In summary, the previous studies on Ng in delirium are few, with relatively small sample sizes and conflicting results, advocating the need for a larger biomarker study exploring the association of Ng with delirium, which is the aim of this study.

5. Methods: clarification of when log transformed values were used vs raw. Rationale for statistical analyses with quantile regression and potential limitations with this method.

> **A. We see that presenting both raw data and transformed data is a bit confusing regarding the interpretation of the results. We have therefore chosen to present only the raw data. We have also altered the order of the presentation in the results section a bit for more clarity for the reader. We have now explained the rationale for statistical analyses with quantile regression in the methods section as mentioned above under point 2.**

6. Probable dementia from IQCODE should be consistent in language and report how they stratify analyses.

> **A. We agree that probable dementia is the best description of the patients in this setting as IQCODE is not a formal diagnosis of dementia, but a surrogate marker. We have adjusted for age, gender and dementia status in our analyses, and have specified this in the methods section, section 2.4 statistical methods. This section now reads:**

Page 10, line 200-202: Further, we used quantile regression to estimate differences in medians between groups and to adjust for sex, age and dementia. Supplementary results are presented for subgroups of dementia status.

7. Clarify delirium diagnostic consensus methods.

> **A. We have added more detail to this description in the methods section of the manuscript, section 2.2 Delirium diagnosis. Both geriatricians considered all the available information for all the patients. Any disagreements were solved through discussion. This section now reads:**

Page 9, line 170-173: Two geriatricians (LOW and BEN) independently considered all the information available for each patient to decide if the DSM-5 criteria (25) were met. Any disagreements were solved through discussion and consensus obtained.

8. Discussion: There are more limitations of this work than reported in the discussion and in the strengths and limitations of this study section after the abstract- including having one CSF sample and attempting to understand numerous associations from one time point and evaluating one post synaptic marker.

A. We agree with your comment. We have updated the discussion section in the revised manuscript and discuss the limitations of having one CSF sample at one time point and that we have evaluated one post synaptic biomarker. We have also added these limitations to the strengths and limitations section after the abstract (see comment 3 in the response to the editor). This section in the discussion now reads:

Page 15, line 339-347: We also want to highlight that we have studied one of many synaptic proteins at one timepoint. It could thus be of interest also to specifically examine the concentrations of other synaptic proteins- e.g. synapse related proteins that have been shown to be downregulated in delirium (21). Ideally biomarker samples should be collected at several timepoints to establish a baseline and register any eventual changes from this baseline after development and resolvement of delirium. It is possible to collect repeated blood samples from acutely admitted patients, but unfortunately both practically and ethically, extremely difficult with repeated CSF sampling over time in such patient groups.

9. The authors do not report how neurogranin levels relate to other delirium biomarkers that have been assessed in this multi-center cohort, as delirium pathophysiology is likely multifactorial, the one marker association with delirium analysis evaluation does not reflect this.

A. We wholeheartedly agree with the reviewer that the pathophysiology of delirium is likely multifactorial. Therefore, we do not believe it is possible to identify a single specific 'delirium biomarker.' This was not the objective of our study. Instead, our rationale for investigating Ng was to use this biomarker to explore the potential role of synaptic dysfunction in the development of delirium

10. Conclusions - The significance of this work should be revisited, including future directions and how finding from this study inform a better understanding of delirium pathophysiology.

A. This is an important point. We have added future directions and how this work better inform our understanding of delirium pathophysiology. Your corrections have helped us improve the manuscript (see the answer to comment 5 to the first reviewer on page 5 of this response for the updated conclusion).

Again, we thank both reviewers and the editor. We would be most grateful if our paper is considered for publication in your journal.

VERSION 2 - REVIEW

**Reviewer**	**2**
**Name**	**Umoh, Mfon E.**
**Affiliation**	**The Johns Hopkins University School of Medicine**
**Date**	**09-Oct-2025**

COI

The authors were very receptive to the reviewers feedback, which have greatly improved the manuscript. The study objectives and outcome, along with the implications have been more clearly addressed with the revisions.

VERSION 2 - AUTHOR RESPONSE

Responses to Reviewer #2:

Reviewer: 2
Dr. Mfon E. Umoh, The Johns Hopkins University School of Medicine
Comments to the Author:

(1) The authors were very receptive to the reviewers feedback, which have greatly improved the manuscript. The study objectives and outcome, along with the implications have been more clearly addressed with the revisions.

> **A. We thank the reviewer for this encouraging comment regarding our manuscript. Your thorough comments and suggestions have helped us improve the quality of the paper.**

Again, we thank both reviewers and the editor. We would be most grateful if our paper is considered for publication in your journal.
